# Healthcare-Associated Infective Endocarditis—Surgical Perspectives

**DOI:** 10.3390/jcm11174957

**Published:** 2022-08-24

**Authors:** Tatjana Musci, Herko Grubitzsch

**Affiliations:** Charité–Universitätsmedizin Berlin, Klinik Für Kardiovaskuläre Chirurgie, Augustenburger Platz 1, 13353 Berlin, Germany

**Keywords:** endocarditis, infective endocarditis, healthcare-associated infective endocarditis, cardiac surgery

## Abstract

Health-care-associated infective endocarditis (HCA-IE), a disease with a poor prognosis, has become increasingly important. As surgical treatment is frequently required, this review aims to outline surgical perspectives on HCA-IE. We searched PubMed to identify publications from January 1980 to March 2022. Reports were evaluated by the authors against a priori inclusion/exclusion criteria. Studies reporting on surgical treatment of HCA-IE including outcome were selected. Currently, HCA-IE accounts for up to 47% of IE cases. Advanced age, cardiac implants, and comorbidity are important predispositions, and intravascular catheters or frequent vascular access are significant sources of infection. Staphylococci and enterococci are the leading causative microorganisms. Surgery, although frequently indicated, is rejected in 24–69% because of prohibitive risk. In-hospital mortality is significant after surgery (29–50%) but highest in patients rejected for operation (52–83%). Furthermore, the length of hospital stay is prolonged. With aging populations, age-dependent morbidity, increasing use of cardiac implants, and growing healthcare utilization, HCA-IE is anticipated to gain further importance. A better understanding of pathogenesis, clinical profile, and outcomes is paramount. Further research on surgical treatment is needed to provide more comprehensive information for defining the most suitable treatment option, finding the optimal time for surgery, and reducing morbidity and mortality.

## 1. Introduction

Infective endocarditis (IE), still a condition with high morbidity and mortality, is a disease that summarizes a broad spectrum of infections of endocardial structures and cardiac implants with multifactorial pathogenesis and therefore requires a collaborative approach for adequate diagnosis and treatment [1,2]. Although community-acquired infective endocarditis (CA-IE) remains the predominant form of the disease, classical predispositions such as congenital or rheumatic valve disease are decreasing and are present in less than 50% of patients today [3]. Due to aging populations, age-depending morbidity, developing healthcare systems, and invasive medical procedures, a significant shift occurred: once a disease of young adults, it now affects older patients who more often develop IE as the result of healthcare-associated procedures. This type of endocarditis is regarded as healthcare-associated infective endocarditis (HCA-IE) and is classically defined as either IE manifesting >48 h after hospital admission or IE associated with a significant invasive procedure performed 6 months prior to clinical diagnosis [4]. HCA-IE, including nosocomial (hospital-acquired) as well as nosohusial infections (acquired in other healthcare settings, i.e., hemodialysis, nursing homes, or day hospitals), is increasingly important and currently accounts for up to 47% of IE cases [5,6,7,8,9]. Furthermore, surgical treatment is frequently indicated but is also associated with characteristic risk in this particular group of patients. Thus, this review aims to outline the surgical perspectives on HCA-IE.

## 2. Methods/Literature Search

**Figure 1:**
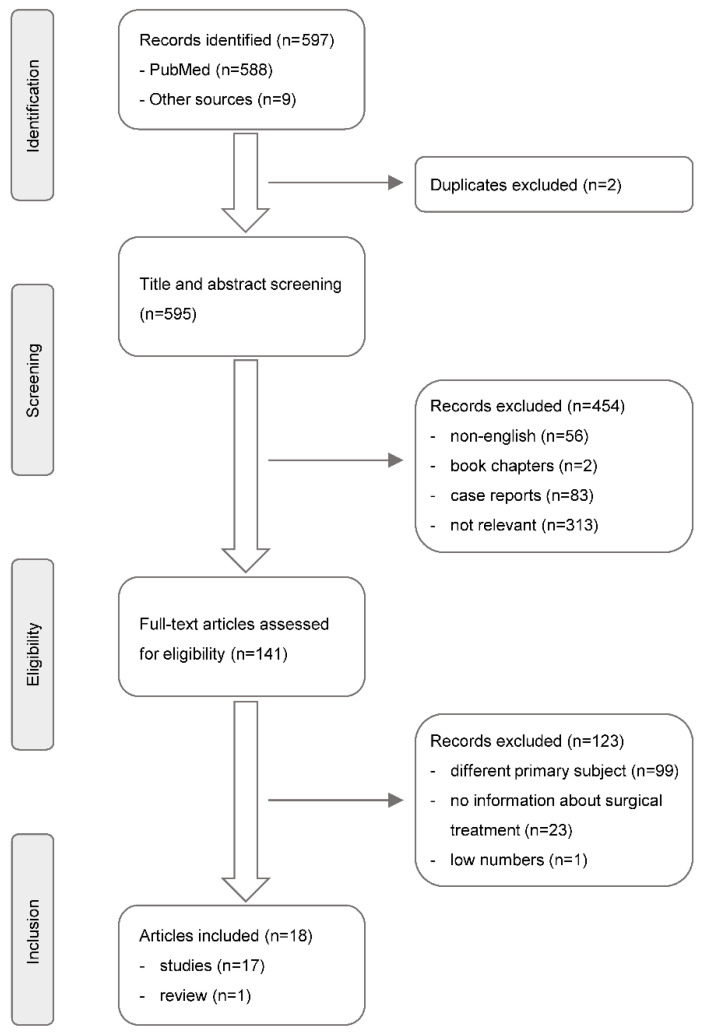
**Evaluation of literature.** After database search and cross-checking of bibliographies (see text), relevant studies were identified following the Preferred Reporting Items for Systematic Reviews and Meta-Analyses (PRISMA) guidelines.

A comprehensive database search was performed on PubMed for the period between 1 January 1980 and 31 March 2022, applying the following search terms: [“infective endocarditis” AND (“health care associated” OR “health care-associated” OR “healthcare associated” OR “healthcare-associated” OR “hospital acquired” OR “nosocomial”)] AND (“cardiac surgery” OR “heart surgery”). Reference lists of other published reviews and relevant reports were cross-checked to identify any additional studies. As depicted in Figure 1, these publications were stepwise evaluated by the authors against a priori inclusion/exclusion criteria to obtain studies that report on surgical treatment of HCA-IE including outcome. Finally, 18 reports were selected after a full-text review.

## 3. Epidemiology and Predisposition

Despite improvements in diagnostic and therapeutic strategies, neither the incidence of IE, ranging between 1.5 and 9.6 cases per 100,000 people, nor its mortality, ranging between 15 and 25%, have decreased [3,6,10]. This is due to simultaneous but opposed trends. The epidemiologic profile of IE, including causative microorganisms, changed, and its complexity increased, especially in developed countries with their characteristics of aging populations and efficient healthcare systems. The proportion of IE classically related to preexisting congenital or rheumatic heart valve disease decreased in favor of cases related to degenerative valvulopathies, prosthetic valves, and cardiovascular implantable electronic devices [3]. The growing importance of HCA-IE results from age-dependent morbidity and healthcare utilization. As listed in Table 1, a lot of predispositions put patients at risk of acquiring IE either in the community or in association with the healthcare system, and a wide spectrum of sources can potentially cause HCA-IE in patients regardless of whether predispositions are present or not. Apart from older age, increased comorbidity, and the increased use of cardiac implants, the necessity of invasive medical procedures increases the risk of bacteremia and bloodstream infections and consequently results in HCA-IE. Thus, intravascular catheters or frequent vascular access (e.g., hemodialysis) are considered the most common source of HCA-IE, accounting for 40–50%, with the peripheral venous catheter being the leading source [4,6,11,12,13,14,15,16].

## 4. Pathophysiological Aspects and Microbiological Findings

The development of IE is multifactorial [1]. Because the normal endocardium is resistant to bacterial colonization, the development of IE requires at least (1) an alteration of the endocardial surface, making it suitable for pathogens to attach and grow as well as (2) the occurrence of a microorganism in the blood, which is - by its quantity and adhering properties - able to colonize. The substrate for bacterial seeding is the secondary thrombotic activation with stratification of thrombotic material on the damaged endocardium (first event), in which bacteria implant during bacteremia (second event) [17]. Further thrombus stratification covers bacteria rendering them less accessible to the immune system (third stage) and favors their rapid multiplication (fourth stage) [17]. Regarding endocardial damage, a significant shift from classical causes, in particular congenital or rheumatic heart disease, to factors, which are related to developed societies and their healthcare systems, occurred during the last century [3]. Thus, the increasing age of the population is not only associated with degenerative valve disease or devices such as heart valve prostheses but also with healthcare-associated procedures increasing in frequency and invasiveness (Table 1). Classically, pathogens gain access to the bloodstream from an oral source, either spontaneously (e.g., implants or chronically infected roots as consequences of advanced cavities) or during a dental procedure (e.g., root canal procedures). In HCA-IE generally, bacteria rather translocate from other epithelial areas depending on the potential source of infection (Table 1). Hospitalization per se, for instance, predisposes to staphylococcal infection originating from the skin due to injection drug use or (infected) intravenous catheters. At the damaged endocardium, the microorganisms proliferate within a protected matrix of platelets and serum molecules, such as fibrin, creating infected vegetations. Apart from detaching vegetation particles that can cause thromboembolic complications and spreading of infection to distant organs, the affected endocardium can be lysed by bacterial products, leading to tissue destructions, predominantly resulting in heart valve regurgitation and/or local abscess formation. Less frequently, vegetations can cause stenotic lesions as well.

Although a variety of microorganisms can cause endocarditis, Staphylococcus aureus, followed by Streptococcus viridans and Enterococcus faecalis, are the most important pathogens being responsible for approximately 80% of IE cases overall [1,10]. Nonetheless, the microbiology of IE varies depending on whether the infection is community-acquired or healthcare-associated. In HCA-IE, staphylococcus species (detected in 55–58%) and enterococci (11–23%) are the causative microorganisms in the vast majority of patients, whereas streptococci (6–24%) seem to play a minor role [4,9,11,12,18]. In infected leads, coagulase-negative staphylococci (e.g., Staphylococcus hominis, Staphylococcus epidermidis) have been recently reported as the predominant infective agents [19]. Importantly, Staphylococcus aureus, known to cause acute, aggressive infections with poor outcomes, is not only a frequent infectious germ in HCA-IE, being detected in 30–45% of cases but also the rate of methicillin-resistant strains (26–47%) is concerning [4,9,11,12,18]. Negative blood cultures, present in 21% of IE overall in the EURO-ENDO cohort, have been reported in less than 8% of HCA-IE studies [4,9,10,11,12,18].

## 5. Diagnostic Challenges and Indications for Surgery

The diagnosis of IE is based on clinical symptoms, blood cultures, and findings of imaging studies, primarily echocardiography [2]. Regarding HCA-IE, it is important to consider that these patients are under medical care for reasons different from IE, where symptoms and signs of IE rather appear secondarily. In general, IE patients present typical signs of IE (Oslers nodes, Janeway lesions, Roth spots) in less than 5% today [10]. If this finding is related to (1) the changing spectrum of causative microorganisms and/or (2) the fact that IE is earlier diagnosed than these distant phenomena can develop remains to be elucidated. Thus, the occurrence of fever, (new) cardiac murmur, and/or laboratory findings of infection, e.g., elevated levels of C-reactive protein (CRP) in patients exposed to the healthcare system for any reason, deserves attention to either exclude or confirm IE, in particular, if predispositions (Table 1) are present. Nevertheless, as exposure to the healthcare system can cause a variety of infections including bacteremia not leading to IE and degenerative heart valve alterations are frequently present in elderly patients, exclusion or confirmation is usually challenging. Clinical suspicion is an important prerequisite for initiating diagnostic workup.

Positive blood cultures and echocardiographic findings (vegetations, abscess, fistula, leaflet perforation, valvular regurgitation, and prosthetic valve dehiscence) remain the cornerstones of diagnosis [2]. In inconclusive cases or possible IE, repeated echocardiography is required, and the addition of further imaging techniques, e.g., cardiac computerized tomography (CT), Fluorine-18-fludeoxyglucose positron emission tomography/computerized tomography (^18^F-FDG PET/CT), magnet resonance imaging (MRI), may provide more reliable results [2,10]. Although the temporal resolution of CT and the spatial resolution of PET are inferior, both appear to be superior in identifying perivalvular complications, particularly important in prosthetic valve endocarditis (PVE). In HCA-IE, indications for surgery, namely heart failure due to valvular dysfunction, embolic manifestations, and persistent infection, are not notably different from CA-IE and can be found in 35–58% of patients [4,12]. Furthermore, there is no evidence that paravalvular complications, e.g., abscess, valvular perforation, or cardiac fistula, occur more frequently in HCA-IE [11]. The diagnosis of cardiac implantable electronic device (CIED) systemic infection or involvement in IE is more challenging, especially without local infection. Once suspicious structures are identified at CIED leads, careful assessment to confirm or rule out endocarditis is needed. Besides serial transthoracic/transesophageal/intracardial echocardiography, ^18^F-FDG PET/CT and radiolabeled leucocyte (white blood cell, WBC) scintigraphy could be helpful, as normal echocardiography does not rule out CIED related infective endocarditis [20]. For clarifying suspected right-sided IE, intracardiac echocardiography is potentially superior to transesophageal echocardiography. Regarding imaging techniques based on ionizing radiation, their specific risks have to be considered. 

For diagnosis as well as for medical and surgical treatment, guidelines strongly recommend a multi-disciplinary approach (endocarditis team) involving cardiologists, cardiac surgeons, specialists for infectious disease, microbiologists, neurologists, neurosurgeons, and other experts, e.g., in congenital heart disease [2].

## 6. Medical Treatment

As in IE in general, successful treatment of HCA-IE also relies on microbial eradication by antimicrobial drugs, preferring more effective bactericidal over bacteriostatic regimens [2]. Treatment of IE should be started promptly after taking three sets of blood cultures. Hence, initial antibiotic therapy is empirical. Empirical treatment, however, is still debated, and its efficacy is less well established by evidence (class IIb recommendations, “may be considered”) [2]. As empirical regimens for HCA-IE or early PVE (defined as prosthetic valve endocarditis occurring within 12 months after valve surgery) should cover methicillin-resistant staphylococci, enterococci, and, ideally, non-HACEK Gram-negative pathogens, vancomycin (30 mg/kg/day i.v. in two doses) with gentamicin (3 mg/kg/day i.v. or i.m. in one dose) is proposed for initial treatment [2]. In healthcare-associated native valve endocarditis, some experts recommend in settings with a prevalence of methicillin-resistant Staphylococcus aureus (MRSA) infections >5%, the combination of cloxacillin (12 g/day i.v. in 4–6 doses) plus vancomycin (30 mg/kg/day i.v. in two doses) until they have the final S. aureus identification [2]. Rifampin (900–1200 mg i.v. or orally in two or three divided doses) is only recommended for PVE, and it should be started 3–5 days later than vancomycin and gentamicin, according to the suggestion of some experts [2]. Once the pathogen is identified, the antibiotic treatment must be adapted to the particular microorganism and its antimicrobial susceptibility pattern. Patients with blood-culture-negative IE should be treated in close collaboration with a specialist for infectious diseases. Additionally, after surgical treatment, antimicrobial therapy has to be continued following the regimen recommended for the causative pathogen [2]. The duration of treatment is based on the first day of effective antibiotic therapy (negative blood culture in the case of initial positive blood culture), not on the day of surgery, and a new full course of treatment should only start if valve cultures are positive, with the choice of antibiotic being based on the susceptibility of the latest recovered bacterial isolate [2].

## 7. Surgical Treatment

**Figure 2:**
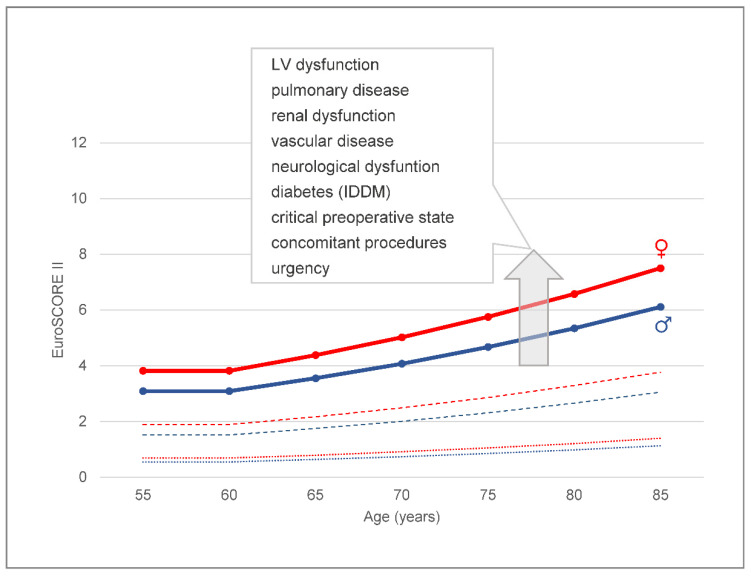
**Operative risk in healthcare-associated infective endocarditis.** The graphs show the calculated risk of mortality (EuroSCORE II) of isolated redo valve replacement due to prosthetic valve endocarditis (PVE) in female (red line) and male (blue line) patients (no further comorbidities) in comparison to isolated redo valve replacement not due to IE (interrupted lines) and isolated primary coronary artery bypass grafting (dotted lines) [http://www.euroscore.org/calc.html, accessed on 22 July 2022]. The insert lists patient- and cardiac-related factors leading to significantly increased risk. For example, in a 73-year-old female patient requiring urgent surgery due to PVE and presenting with end-stage renal failure and moderate LV dysfunction, the calculated risk of mortality is 17%. IDDM, insulin-dependent diabetes mellitus.

The goals of surgical treatment are radical resection of infectious material, prevention of embolism, and restoration of valvular function. In general, surgery is performed in about 50% of all patients with IE, although a theoretical indication is present in 70% [2,10]. Remarkably, the rate of HCA-IE patients undergoing surgical treatment tends to be lower and varies considerably (Table 2). It was reported that surgery, although indicated, was rejected in 24–69% because of prohibitive perioperative risk [4,12,21]. Thus, significantly fewer patients with HCA-IE undergo surgical treatment compared to CA-IE [4,9,11,12,18]. For several reasons, the risk of surgery is higher in HCA-IE. Older age, infection of cardiovascular implants, e.g., heart valve prostheses, and foremost age-dependent comorbidity are important factors for increased mortality (Figure 2). Endocarditis per se as well as re-operations are also significant predictors of mortality [22]. Moreover, the high rate of Staphylococcus aureus infection turns HCA-IE into an acute disease frequently requiring urgent surgery, another predictor of mortality [23]. Regarding urgency, less than a quarter has been electively performed procedures in HCA-IE [4,12,18]. Commonly performed surgical techniques, namely valve replacement or repair, debridement, and reconstruction of infected tissue, as well as transvenous or open chest removal of infected cardiac electronic devices, do not differ from other IE entities. Even in patients without definite involvement in the CIED system, complete hardware removal is recommended [20]. Regarding the transvenous extraction of infected leads, the persistence of infected masses (ghosts) should also be considered [24]. Although prosthetic valves are more frequently affected in HCA-IE as compared to CA-IE, approaching up to 29%, it is unclear if more repeat valve operations are performed accordingly [9,12]. Regarding concomitant procedures, it needs to be elucidated if the operative risk can be reduced when alternative catheter-based interventions are chosen. As outlined above, antibiotic treatment has to be continued postoperatively and may be adapted to intraoperative findings. 

## 8. Outcomes

In HCA-IE, outcomes, foremost survival, are significantly inferior as compared to CA-IE. In-hospital mortality, being 18–68% overall (Table 2), is highest in patients rejected for theoretically indicated surgery ranging between 52% and 83% [4,9,12,18,27]. Nonetheless, the theoretical benefit of surgical treatment has to be balanced against its risks. Up to 50% of in-hospital mortality is also remarkably increased after surgery, as summarized in Table 2. Older age and comorbidity, including IE complications such as acute organ failure and/or septic shock, essentially explain the unfavorable course either with or without surgery and give furthermore reasons why surgical treatment is often refused. Moreover, it was reported that complications such as heart failure, renal failure, and septic shock occurred more frequently in HCA-IE and that hospital stay, an indicator for resource utilization, was prolonged [4,9,18]. Regarding long-term outcomes, the risk of death persists after early treatment of HCA-IE, and mortality at one year was reported to be 35–60% [12,30].

## 9. Limitations

The study populations of included references are very different, i.e., different countries/continents, different centers, different patient cohorts, different study periods, etc. This heterogeneity among studies is an important limitation as it prevents the pooling of collective key characteristics and outcomes for analysis. In terms of study design, there is no publication comparing the surgical and medical treatment of HCA-IE. Nonetheless, using the semi-systematic approach as transparently described provides a current understanding of this complex area and shines a light on issues that warrant further research. Although the data regarding epidemiological features of HCA-IE refer to well-designed epidemiological studies on a more global level, they are still scarce and have limitations (incomplete case ascertainment, use of varying case definitions, referral bias, over-representation of developed countries, and a shortage of population denominators), to which barely cover a period beyond a decade.

## 10. Conclusions

In summary, HCA-IE, characterized by high complication rates and adverse outcomes, continues to gain importance. Despite effective preventive measures, e.g., antimicrobial prophylaxis in patients at risk, meticulous care of intravascular lines, etc., its incidence is anticipated to rise, basically due to the increasing use of invasive medical procedures in the aging population. Moreover, the high rate of staphylococcal infection rather turns HCA-IE into an acute disease. Diagnosis, risk assessment, and decision making remain challenging. Although the theoretical indications for surgery are clear, their practical application relies largely on the clinical status of the patient, the patient’s comorbidities, and the patient’s operative risk. Considering that no single operative risk score is perfect, preoperative assessment of operative risk is of utmost importance. Given the paucity of published data, further research on surgical treatment of HCA-IE is needed to provide more comprehensive information that can support defining the most suitable treatment option, finding the optimal time for surgery, and reducing morbidity and mortality. 

## Figures and Tables

**Table 1:** **Predispositions of infective endocarditis and sources of infection in healthcare-associated infective endocarditis**.

Predisposition	Potential Source of Infection
**Prosthetic heart valve** * Congenital heart disease * Previous IE * **Valvular heart disease** **Cardiac electronic device** Skin infection Chronic (auto-)immune disease Immunosuppressive therapy Long-term corticoid therapy Cancer/chemotherapy iv. drug dependency Chronic alcohol abuse Residence in a nursing home or long-term care facility	Dental procedures * (Central) venous catheter/chronic iv. access **Chronic hemodialysis** **Hospital/ICU stay** Invasive procedures: Respiratory tract procedures Gastrointestinal procedures Genitourinary procedures Dermatological procedures Musculoskeletal procedures Cardiovascular surgery Cardiac interventions Vascular interventions

Adapted from references [2,3,9,11,12]. The most common predispositions and sources of infection are marked in bold. * according to present guidelines [2], antibiotic prophylaxis is only recommended for dental procedures requiring the manipulation of the gingival or periapical region of the teeth or perforation of the oral mucosa in patients with cardiac conditions with the highest risk of infective endocarditis (prosthetic heart valve including any prosthetic material used for valvular repair, congenital heart disease, and previous IE).

**Table 2:** **Surgical treatment in healthcare-associated infective endocarditis**.

Reference	Study Period	Patients with HCA-IE n (%)	Age (Median) yrs	Surgery Performed %	Postoperative In-Hospital Mortality %	Overall HCA-IE In-Hospital Mortality %
Terpenning et al. [25]	1976–1985	22 (14.3)	55 *	54.5	41.7	40.9
Chen et al. [26]	1979–1991	30 (16.8)	62	26.6	37.5	40.0
Lamas et al. [15]	1985–1996	22 (14)	51.4 *	27.3	0	50.0
Gouëllo et al. [21]	1992–1997	22 (100)	65 *	22.7	40.0	68.2
Martin-Davila et al. [14]	1985–1999	38 (7.7)	49.6 *	42.1	-	26.3
Giannitsioti et al. [27]	2000–2004	42 (21.5)	64.5	17.9	-	39.5
Fernandez-Hildago et al. [12]	2000–2007	83 (28.4)	65.3	22.9	47.4	45.8
Benito et al. [11]	2000–2005	557 (34)	63	41.0	-	25.0
Rogers et al. [28] ^a^	1991–2006	26 (96.3)	64	59.0	44.0	66.0
Lomas et al. [4]	1984–2007	127 (16.0)	60.1 *	44.1	43.1	44.9
Sy et al. [18]	2000–2006	463 (43.2)	68	19.0	-	22.0
Francischetto et al. [29]	2006–2011	53 (35.1)	47.2 *	64.0	29.0	32.0
Yang et al. [23]	1992–2012	28 (18.8)	43.5	57.1	-	17.9
Garrido et al. [30] ^b^	2006–2016	26 (25)	46.5 *	46.2	50.0	38.5
Hwang et al. [7]	2000–2014	121 (21.6)	51.3 *	38.0	-	27.3
Kiriyama et al. [9]	2007–2018	53 (33.5)	72	41.5	-	32.1
Pericas et al. [16] ^c^	2000–2006 2008–2012	558 (8.3)	59.9	30.6	31.5	30.4

* mean, ^a^ only patients with IE caused by MRSA (n = 27), ^b^ nested case-control study, early PVE versus control (valve replacement without PVE), ^c^ only patients receiving chronic hemodialysis.
